# Bees of the Azores: an annotated checklist (Apidae, Hymenoptera)

**DOI:** 10.3897/zookeys.642.10773

**Published:** 2017-01-03

**Authors:** Julie A. Weissmann, Ana Picanço, Paulo A.V. Borges, Hanno Schaefer

**Affiliations:** 1 Technical University of Munich, Dept. Ecology & Ecosystem Management, Plant Biodiversity Research, Emil-Ramann Str. 2, D-85354 Freising, Germany; 2 CE3C – Centre for Ecology, Evolution and Environmental Changes/Azorean Biodiversity Group and University of Azores, Faculty of Agriculture and Environment, Rua Capitão João d´Ávila, Pico da Urze, 9700-042 Angra do Heroísmo, Terceira, Açores, Portugal

**Keywords:** Apidae, Azores, Hylaeus azorae, Hymenoptera, pollination

## Abstract

We report 18 species of wild bees plus the domesticated honeybee from the Azores, which adds nine species to earlier lists. One species, *Hylaeus azorae*, seems to be a single island endemic, and three species are possibly native (*Colletes eous*, *Halictus villosulus*, and *Hylaeus pictipes*). All the remaining bee species are most likely accidental introductions that arrived after human colonization of the archipelago in the 15^th^ century. Bee diversity in the Azores is similar to bee diversity of Madeira and Cape Verde but nearly ten times lower than it is in the Canary Islands.

## Introduction

**Figure 1.**
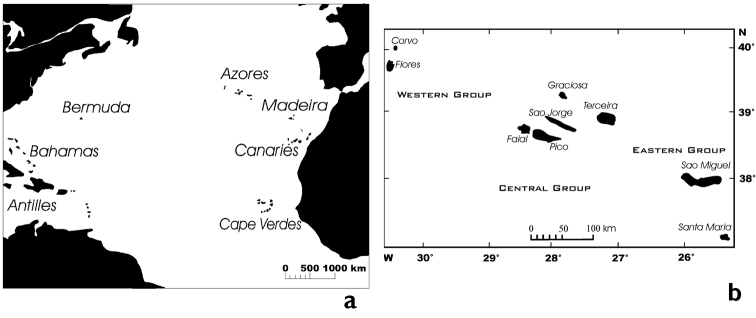
**a** Map of the Northern Atlantic islands **b** Azores archipelago (modified from Schaefer et al. 2011).

The Azores are an archipelago of nine islands and several small islets which range in age from 5.5 million years for Santa Maria (Feraud et al. 1981) to 0.27 million years for Pico (Demand et al. 1982). They are in a very isolated position in the North Atlantic, c. 1300 km from the European continental coast, 1900 km from Newfoundland and 880 km from Madeira, the closest archipelago (Fig. 1). It is therefore not surprising that a strictly terrestrial insect group such as the bees (Hymenoptera, Apidae) is represented with a low number of lineages in the Azores. According to the latest published checklist (Franquinho de Aguiar et al. 2010) only nine species plus the honeybee (*Apis mellifera*) occur in the archipelago. The latter is kept by apiculturists on most islands but currently not known to exist in the wild on any of the Azorean islands. Even though the number of species is low, wild bees are relatively common in the Azores and can occur in high numbers of individuals in natural vegetation, often visiting endemic plant species like the Azores goldenrod (*Solidago azorica*, Asteraceae) or Azores bellflower (*Azorina vidalii*, Campanulaceae) (Weissmann and Schaefer, unpubl. data), but also exotic invasive plant species (Picanço et al., unpubl. data). Their importance for pollination, a key ecosystem service, and thus for survival of the endemic flora and crop production in the Azores has not been studied very well so far. As mentioned by Borges et al. (2010), Hymenoptera in general are poorly studied in the Azores, and there is the urgent need to address the Linnean shortfall and carry out basic inventory and taxonomic work, later followed by more advanced ecological and behavioural studies.

One of the reasons for the lack of pollinator studies in the Azores could be their difficult taxonomy and the challenge to identify wild bee species in the field. Our aim therefore is to provide a comprehensive up-to-date checklist of the bee species of the Azores and highlight characters suitable for field identification, without the need to collect the bee and examine e.g. the male genital apparatus or micropunctures on the scutum. We try to avoid jargon and complicated technical terms as much as possible to allow ecologists and botanists who are not experienced entomologists to identify all bees of the Azores to species- or at least genus-level. In this way, we hope to stimulate more research on bees and pollinators in general in the archipelago, in order to improve conservation management of endangered plants as well as fruit production.

## Material and methods

To obtain a comprehensive species list, we combined observation data of almost two decades of fieldwork by all authors with a review of the available literature, discussions with many experts (see acknowledgements) and visits to the bee collections in Linz (Biology Center, Oberösterreichisches Landesmuseum Linz) and Munich (Bavarian State Collection of Zoology, ZSM). Most of our field work was done on Corvo, Flores, Faial, Graciosa, Santa Maria, and Terceira, with occasional bee observations on the three remaining islands of the archipelago. We made thousands of bee observations during all these years but did not collect large numbers of voucher specimens. Instead, we just took a few selected individuals every year, often animals found dead on roadsides, in spider webs or in Japanese beetle traps (Azores Agriculture Services monitoring plan for *Popillia japonica*). These were identified using the latest morphological identification keys for Europe (e.g., Amiet 1996, Amiet et al. 2001, Amiet et al. 2004, Amiet and Krebs 2014, Falk and Lewington 2015, Scheuchl and Willner 2016). We tried to obtain photographs of living individuals in addition to the voucher specimens, since these are an important source of ecological and behavioural information (e.g., flower visitation, phenology, nesting and mating behaviour) not available from collected material.

For all species, except *Apis mellifera*, *Bombus pratorum*, *Halictus lativentris*, *Hylaeus azorae*, *Megachile concinna*, *Megachile pyrenaica*, and *Osmia niveata*, we used DNA barcoding to obtain some sort of molecular confirmation of the species identity. We extracted DNA from legs or the thorax of up to six dry or ethanol-preserved individuals per species using a NucleoSpin tissue kit (Macherey-Nagel, Germany) following the manufacturer’s protocol. Then we used polymerase chain reaction (PCR) to amplify c. 600 nucleotides of the mitochondrial cytochrome c oxidase I (COI) gene as described in Schmidt et al. (2015). For PCR we relied on the Kapa2G Fast ready mix (Kapa Biosystems, Wilmington, USA) following the suggested standard protocol. Following Smith et al. (2012), we first worked with the primer combination LepF1 and LepR1, and for poor quality DNA samples added the internal primers C_ANTMR1D and RonMWASPdeg_t1. Because of problems with *Wolbachia* (see below), we designed a new forward primer Hym-COI-F (TAA GAA TAA TTA TTC GWA TAG AAT TAA G), which was used together with LepR1 for all halictid and colletid bees. We cleaned the PCR products enzymatically with ExoSAP-IT mix (Affymetrix, Santa Clara, USA) and sent them to a private company (GATC, Konstanz, Germany) for Sanger sequencing. We edited and assembled the raw sequence reads using Geneious 6.1.8 (Biomatters, Auckland, New Zealand). Then we performed BLAST (Basic Local Alignment Search Tool) searches in GenBank (www.ncbi.nlm.nih.gov/genbank/) to compare our sequences to all publicly available bee sequences. Furthermore, we aligned the obtained COI sequences with additional sequences from other parts of the species ranges downloaded from GenBank in Geneious 6.1.8 using the Geneious alignment algorithm. We added COI sequences from GenBank of the six species, for which no sequence from Azorean material was available and *Vespula germanica* (introduced invasive in the Azores) as an outgroup and used RAxML v. 8.1.18 (Stamatakis 2014) on the CIPRES Science Gateway v.3.3 (http://phylo.org) to estimate a maximum likelihood (ML) phylogeny.

## Results

In total, we found evidence for occurrence of 18 wild bee species from seven genera (*Anthidium*, *Bombus*, *Colletes*, *Halictus*, *Hylaeus*, *Megachile*, and *Osmia*) and four families (Apidae, Colletidae, Halictidae, and Megachilidae) plus the domesticated honeybee *Apis mellifera* in the Azores. For 15 species, we have detailed photographs from the field in the Azores and for 17 species, at least one specimen from the Azores exists in our collections or in other public collections. For one species, *Osmia niveata*, we found only literature data (see species accounts for details).

**Figure 2.**
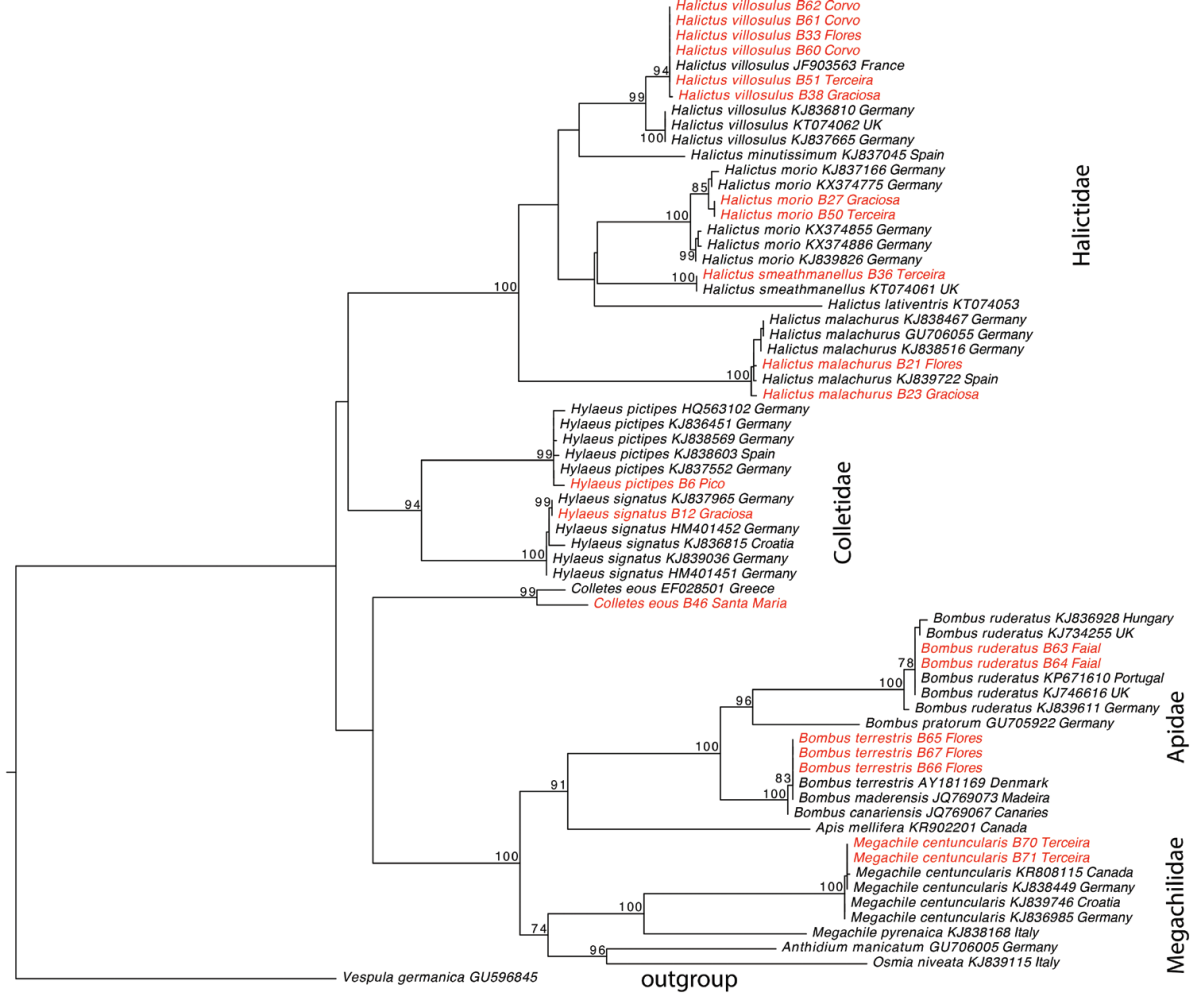
ML phylogeny estimate of the bee species of the Azores archipelago based on 657 nucleotides of the COI gene; sequences of Azorean samples in red, those from other regions in black; the phylogeny is rooted on the Azorean invasive *Vespula germanica*; for sequences downloaded from GenBank, the accession numbers are given, for new Azorean sequences we give the collection number of TUM; the two species *Megachile concinna* and *Hylaeus azorae* are missing in the tree because of lack of material from the Azores for sequencing and lack of sequences from other sources.

We managed to produce COI sequence data for ten species, which we used for comparison with GenBank sequences, mostly those from the study of Schmidt et al. (2015). A match of 98-100% to at least two GenBank sequences with the same species identity was interpreted as support of our morphological identification (see species accounts for exceptions). DNA extraction from both ethanol and air dried specimens of different age (up to 17 years) was successful. However, we observed that PCR reactions with the standard barcoding primer combination LepF1-LepR1 resulted mostly in sequences of *Wolbachia* parasites, whereas the use of internal primers or our more specific newly designed forward primer gave 100% bee sequences (see also Smith et al. 2012). All sequences have been deposited in GenBank (acc. no. KX824760-80). The ML community phylogeny (Fig. 2) is well resolved and all genera and species are recovered as monophyletic, with very little intraspecific genetic variation. Four of the three families are also recovered as monophyletic. The only exception is Colletidae, where the *Hylaeus* lineage and the *Colletes* lineage do not form a clade. However, the bootstrap values are very low at these nodes and it should be kept in mind that all this is based on just 657 nucleotides of the mitochondrial COI gene. To obtain a more robust phylogeny estimate, additional mitochondrial and nuclear DNA sequences would be necessary but this is beyond the scope of our checklist.

The current state of knowledge regarding the distribution per island within the archipelago is shown in Table 1. Most species (14) are known from the largest island, São Miguel, followed by Faial with 12 species and the small islands Corvo and Flores with 11 species each. Only seven species are currently known from São Jorge. Most species seem to occur on several or all islands. Just one species, *Hylaeus azorae*, might be a single-island endemic, known only from Pico island. *Megachile pyrenaica* and *Bombus pratorum* are also restricted to a single island, Santa Maria and Faial respectively, but certainly recent introductions. *Megachile concinna* is known only from the islands of the western group, *Colletes eous* only from the eastern group.

**Table 1.** Species list with distribution per island and presumed status in the archipelago.

Species	Corvo	Flores	Faial	Pico	Graciosa	S. Jorge	Terceira	S. Miguel	Sta. Maria	Status
*Anthidium manicatum* (Linnaeus)			X					X		introduced
*Apis mellifera* Linnaeus	X	X	X	X	X	X	X	X	X	domesticated
*Bombus pratorum* (Linnaeus)			X							introduced
*Bombus ruderatus* (Fabricius)	X	X	X	X	X	X	X	X	X	probably introduced
*Bombus terrestris* (Linnaeus)	X	X	X	X	X	X	X	X	X	probably introduced
*Colletes eous* (Morice)								X	X	possibly native
*Halictus lativentris* (Schenk)			X					X		probably introduced
*Halictus malachurus* (Kirby)	X	X	X	X	X	X		X	X	probably introduced
*Halictus minutissimus* (Kirby)			X				X	X		probably introduced
*Halictus morio* (Fabricius)	X	X		X	X	X	X	X	X	probably introduced
*Halictus smeathmanellus* (Kirby)	X	X		X			X	X		probably introduced
*Halictus villosulus* (Kirby)	X	X	X	X	X	X	X	X	X	possibly native
*Hylaeus azorae* (Warncke)				X						**endemic**
*Hylaeus pictipes* (Nylander)	X	X	X	X	X			X		possibly native
*Hylaeus signatus* (Panzer)	X	X	X	X	X		X	X	X	introduced
*Megachile centuncularis* (Linnaeus)	X	X	X			X	X	X		probably introduced
*Megachile concinna* (Smith)	X	X								introduced
*Megachile pyrenaica* Lepeletier									X	introduced
*Osmia niveata* (Fabricius)							?	?		doubtful

Our current state of data with regards to foraging is summarized in Table 2. It is based on the observations over the past decades but is certainly not exhaustive. For several species, e.g. *Bombus pratorum*, a much wider host plant spectrum can be assumed. Only one species, *Hylaeus signatus*, is known to be a specialist in other parts of its range but it seems to be less strict in the Azores (see below). Foraging preferences of four species (*Megachile pyrenaica*, *Halictus lativentris*, *Hylaeus azorae*, *Osmia niveata*) are unknown in the Azores.

**Table 2.** Flowering plant groups visited by wild bee species in the Azores.

**Bee species**	**Plant family**
*Anthidium manicatum*	Asteraceae, Fabaceae, Lamiaceae
*Bombus pratorum*	Lamiaceae
*Bombus ruderatus*	Aizoaceae, Asteraceae, Boraginaceae, Brassicaceae, Elaeagnaceae, Ericaceae, Fabaceae, Hydrangeaceae, Hypericaceae, Lamiaceae, Myrtaceae, Plantaginaceae, Polygonaceae, Primulaceae, Resedaceae, Rosaceae, Scrophulariaceae, Solanaceae, Tropaeolaceae, Verbenaceae, Zingiberaceae
*Bombus terrestris*	Aizoaceae, Asteraceae, Boraginaceae, Brassicaceae, Campanulaceae, Caprifoliaceae, Elaeagnaceae, Ericaceae, Frankeniaceae, Iridaceae, Lamiaceae, Myrtaceae, Pittosporaceae, Plantaginaceae, Primulaceae, Resedaceae, Rosaceae, Solanaceae, Tropaeolaceae, Zingiberaceae
*Colletes eous*	Asteraceae
*Halictus lativentris*	Unknown
*Halictus malachurus*	Apiaceae, Asteraceae, Campanulaceae, Ericaceae, Resedaceae, Tamaricaceae
*Halictus minutissimus*	Asteraceae (*Erigeron karvinskianus*)
*Halictus morio*	Apiaceae, Asteraceae, Campanulaceae, Fabaceae, Hydrangeaceae, Hypericaceae, Lamiaceae, Lythraceae, Oxalidaceae, Polygonaceae, Ranunculaceae, Rosaceae, Scrophulariaceae, Zingiberaceae
*Halictus smeathmanellus*	Asteraceae (*Solidago azorica*), Campanulaceae (*Azorina vidalii*), Fabaceae
*Halictus villosulus*	Asteraceae, Ericaceae, Fabaceae, Frankeniaceae, Gentianaceae, Hydrangeaceae, Hypericaceae, Lamiaceae, Plantaginaceae, Primulaceae, Rosaceae
*Hylaeus azorae*	Unknown
*Hylaeus pictipes*	Asteraceae (*Solidago azorica*, *Helminthotheca echioides*), Ranunculaceae (*Ranunculus cortusifolius*)
*Hylaeus signatus*	Resedaceae (*Reseda luteola*), Tamaricaceae (*Tamarix africana*)
*Megachile centuncularis*	Asteraceae, Fabaceae
*Megachile concinna*	Fabaceae (*Lotus corniculatus*)
*Megachile pyrenaica*	Unknown
*Osmia niveata*	Unknown

## Annotated checklist

In the following, we provide short species accounts for all 19 bee species for which we found evidence that they exist or have existed in the Azores. General distribution data and most information on nesting and social behaviour is based on literature (Amiet and Krebs 2014, Falk and Lewington 2015, Scheuchl and Willner 2016). Information on foraging and phenology is based on observations by the authors unless other sources are indicated. This information was compiled during fieldwork in the Azores since 1998 and is therefore much more comprehensive than the data from the collected individuals. The specimen numbers refer to the collections at Technical University of Munich (TUM) and University of Azores, Entomoteca Dalberto Teixeira Pombo (EDTP).

### Apidae

#### *Bombus* Latreille

Large, hairy, predominantly black, yellow- or white-banded eusocial bees. Nests are built in cavities in the ground, preferably old mouse nests. Females collect pollen with special structures, pollen baskets, on the tibiae of their hind legs. These are absent in parasitic bumblebees of which none are known in the Azores. Three species in the Azores.

##### Bombus pratorum

Taxon classificationAnimaliaHymenopteraApidae

(Linnaeus)

###### Description.

**Figure 3.**
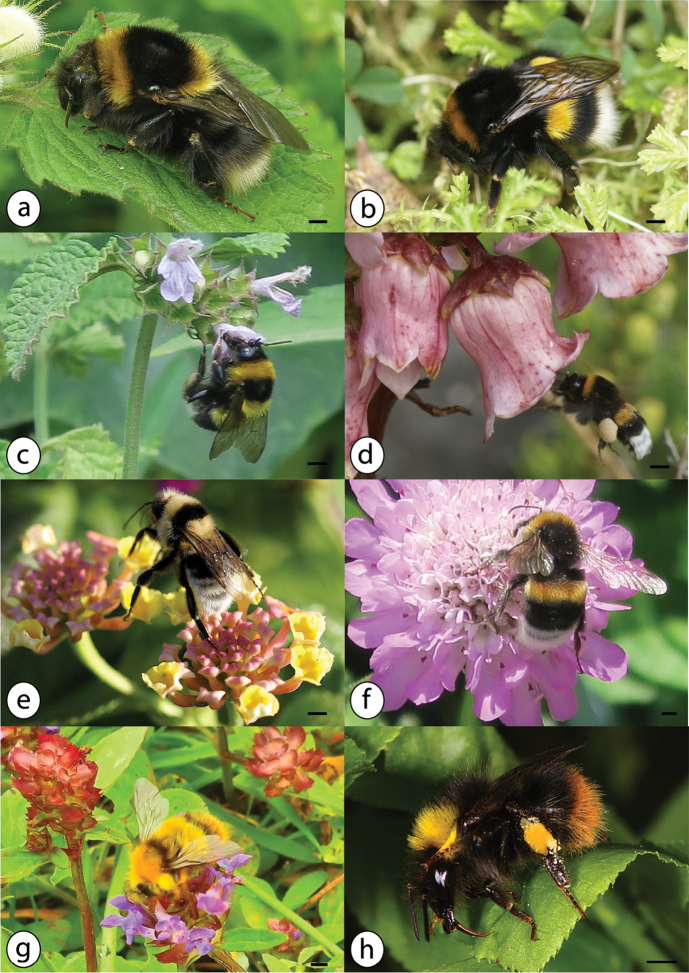
Azorean *Bombus* species: **a** *Bombus ruderatus* queen, UK **b** *Bombus terrestris* queen, July 2016, Terceira; c) *Bombus ruderatus* worker on *Ballota nigra* (Lamiaceae), July 2016, Terceira (Angra do Heroísmo) **d** *Bombus terrestris* worker on *Azorina vidalii* (Campanulaceae), July 2015, Corvo **e** *Bombus ruderatus* male on *Lantana camara* (Verbenaceae), September 2010, São Miguel **f** *Bombus terrestris* male, July 2016, Flores **g** *Bombus pratorum* male on *Prunella vulgaris* (Lamiaceae), August 2015, Faial (Miradoura da Caldeira) **h** *Bombus pratorum* worker, Norway; photos: Steven Falk (**a**), Hanno Schaefer (**b, c, e, f**), Julie A. Weissmann (**d**), Valter Jacinto (**g**) and Kjetil Fellheim on wikipedia (creative commons license) (**h**); scale bars 2 mm.

Large black bee (wing length 13 mm in queens and 10 mm in workers and males), queens and workers with a bright yellow band on the thorax close to the head, a second one in the middle of the abdomen on tergite 2 (often paler or missing in workers), and orange tip of the abdomen; males with yellow head, large bright yellow band in the upper part of the abdomen, and orange tip of the abdomen (Fig. 3g–h).

###### Distinguishing features.

Smallest bumblebee of the Azores and only bee species with orange tip of abdomen in the archipelago.

###### General distribution.

From Portugal in the West to Kamchatka in the East, and from northern Scotland in the North to northern Iran in the South.

###### Distribution in the Azores.

Faial.

###### First record.

2015.

###### Nesting.

No nests observed in the Azores, elsewhere the species has colonies of 50-120 individuals in existing cavities on the surface or in the soil.

###### Social behaviour.

Primitively eusocial.

###### Foraging.

The only observation in the Azores was on *Prunella vulgaris* (Lamiaceae), elsewhere the species is polylectic.

###### Phenology.

August.

###### Material.

None.

###### Note.

One single observation by Valter Jacinto on Faial Island, Miradouro da Caldeira (c. 850 m asl.), 13 August 2015, (http://www.inaturalist.org/observations/1910834).

##### Bombus ruderatus

Taxon classificationAnimaliaHymenopteraApidae

(Fabricius)

###### Description.

Large black bee; wing length 18 mm and total length up to 22 mm in queens, workers with wing length of 13 mm and total length up to 16 mm, males with wing length of 14 mm; queens and workers with two brownish-yellow bands on the thorax, a narrow yellow band and white tip of the abdomen (Fig. 3a, c); males with paler yellow bands, more white abdomen and white hairy face pattern (Fig. 3e).

###### Distinguishing features.

Of the two bumblebee species in the archipelago, *Bombus ruderatus* is the paler species and can be recognized by the different colour pattern (two yellow bands on the thorax vs. one yellow thorax band in *Bombus terrestris*).

###### General distribution.

Madeira; throughout Europe from the Iberian Peninsula to southern Scandinavia; North Africa; Asia to Siberia in the East; introduced and invasive in New Zealand and South America (Chile, Argentina).

###### Distribution in the Azores.

All islands.

###### First record.

1865 (Godman 1870, as *Bombus hortorum*).

###### Nesting.

Colonies of up to 50-100 workers in existing holes in the ground.

###### Social behaviour.

Primitively eusocial.

###### Foraging.

Polylectic, visits a wide range of species, including exotic invaders like *Lantana camara*, Verbenaceae (Fig. 3e).

###### Phenology.

All year.

###### Material.

Faial (Horta), August-September 1930, leg. L. Chopard, det. Benoist (Benoist et al. 1936, not seen) Faial (Castello Branco, from Japanese beetle trap), 1999, 1 worker, 9 males, leg. H. Schaefer, coll. TUM (specimens B48-B49, B52-B56, B63-B64, B72).

COI sequences of specimens B63-64 (TUM), acc. no. KX824771-72, are identical to *Bombus ruderatus* sequences from Portugal and UK in GenBank (see Fig. 2).

###### Note.

Reports of *Bombus hortorum* L. by Benoist et al. (1936) refer to this species.

##### Bombus terrestris

Taxon classificationAnimaliaHymenopteraApidae

(Linnaeus)

###### Description.

Large black bee (wing length 18 mm, total length 20–22 mm in queens; wing length 13 mm, total length 11–17 mm in workers; wing length 14 mm, total length 11–17 mm in males) with one strong brownish-yellow band on the thorax close to the head, a second one in the middle of the abdomen (tergite 2), and bright white tip of the abdomen (Fig. 3b, d, f).

###### Distinguishing features.

In contrast to *Bombus ruderatus*, only one yellowish band on the thorax, which is deeper in colour than in the previous species.

###### General distribution.

North Africa, Eurasia (Portugal to Norway in the North and Japan in the East), populations on the Canaries and Madeira have been described as distinct species but at least for Madeira, this is not supported (Widmer et al. 1998, Williams et al. 2012); introduced and invasive in parts of Asia, New Zealand, Tasmania, and South America.

###### Distribution in the Azores.

All islands.

###### First record.

Williams et al. 2012 (no island specified).

###### Nesting.

Colonies of several 100 workers in existing holes in the ground.

###### Social behaviour.

Primitively eusocial.

###### Foraging.

Polylectic, visits a wide range of species, including endemics like *Azorina vidalii* (Campanulaceae, Fig. 3d), and invasive exotics like *Hedychium gardnerianum* (Zingiberaceae) and *Leycesteria formosa* (Caprifoliaceae).

###### Phenology.

All year (queens flying December/January to March).

###### Material.

Graciosa, 1 worker; Flores (Santa Cruz and Lajes das Flores, from Japanese beetle trap), July 2016, 9 queens, 9 workers, all leg. H. Schaefer, coll. TUM (specimens B2, B47, B65-B67, B73-B87).

COI sequences of specimens H. Schaefer B65-B67 (TUM), acc. no. KX824773-75, are identical to a *Bombus terrestris* sequence from Denmark and a *Bombus maderensis* sequence from Madeira in GenBank but differ slightly from *Bombus canariensis* JQ769067 (see Fig. 2).

### Colletidae

#### *Colletes* Latreille

Medium-sized solitary bees. Most species have conspicuous bright hair bands on the abdomen. Nests are built in light soil, often in aggregation. The chambers are lined with a cellophane-like substance. Females collect pollen on the hind legs and the sides of the propodeum. One species in the Azores.

##### Colletes eous

Taxon classificationAnimaliaHymenopteraColletidae

(Morice)

###### Description.

**Figure 4.**
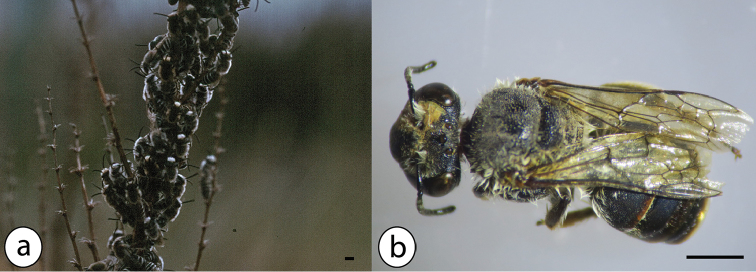
Azorean *Colletes* species: **a** aggregation of more than 50 males of *Colletes eous*, June 2001, Santa Maria (pasture near airport) **b** *Colletes eous* female from Santa Maria (B46, TUM); photos: Hanno Schaefer (**a**) and Julie A. Weissmann (**b**); scale bars 2 mm.

Medium-sized bee (total length 10–13 mm in both sexes, wing length c. 8 mm in females) with orange brown hairy thorax and dark abdomen with white hairy tergite margins (Fig. 4a–b).

###### Distinguishing features.

Medium-sized bee with light brown thorax and conspicuously black and white ringed abdomen.

###### General distribution.

Mediterranean region to Central Asia (Kuhlmann 2005).

###### Distribution in the Azores.

São Miguel and Santa Maria.

###### First record.

1972.

###### Nesting.

See above.

###### Social behaviour.

Solitary.

###### Foraging.

Based on literature polylectic.

###### Phenology.

June-July.

###### Material.

Santa Maria (Vila do Porto), July 1972, 1 male, leg. N.L.H. Krauss, coll. Snow Entomological Museum, Lawrence, Kansas; São Miguel (Mosteiros), 24.07.2004, 1 male, leg. A. Kroupa, coll. Kroupa (M. Kuhlmann, pers. comm., 28.09.2016); Santa Maria, July 2001, 1 female, leg. H. Schaefer, det. M. Kuhlmann, coll. TUM (B46).

###### Note.

According to M. Kuhlmann, Kiel (pers. comm., 23.11.2015) earlier reports of *Colletes canescens* Smith 1853, from the islands, most likely refer to this species.

DNA barcoding of specimen B46 (acc. no. KX824776) and BLAST search in GenBank resulted in *Colletes eous* from Greece as best match (acc. no. EF028501). However, the similarity is only 94%, which means that there are 23 nucleotide differences distributed over the entire COI sequences (see also branch length difference in the ML tree, Fig. 2). This suggests that the Azorean *Colletes* is not the same species as the one sequenced from Greece but more morphological and genetic data is needed to solve this question.

#### *Hylaeus* Fabricius, (*Prosopis*)

**Figure 5.**
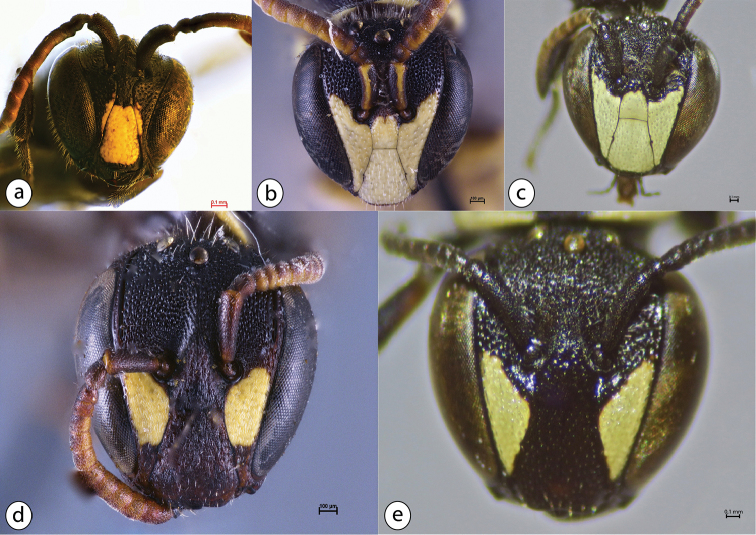
Face patterns of the Azorean *Hylaeus* species; males in the top row, females below: **a** *Hylaeus azorae* **b** *Hylaeus pictipes* **c** *Hylaeus signatus* **d** *Hylaeus pictipes* **e** *Hylaeus signatus*; photos: Esther Ockermüller (Oberösterreichisches Landesmuseum Linz) (**a, b, d**) and Julie A. Weissmann (**c, e**); scale bars 0.1 mm.

All Azorean *Hylaeus* species are small black bees with whitish or yellow face patterns, the males with a mask-like pattern (Fig. 5a–c), the females usually with two dots (Fig. 5d–e). They are solitary bees and build their nests in hollow twigs or other cavities in wood, stone or vertical soil banks. Females collect pollen in their crop. Three species in the Azores, none of them mentioned in the previous lists for Macaronesia (Erlandsson 1983) or the Azores (Franquinho de Aguiar et al. 2010).

##### Hylaeus (Prosopis) azorae

Taxon classificationAnimaliaHymenopteraColletidae

(Warncke)

###### Description.

**Figure 6.**
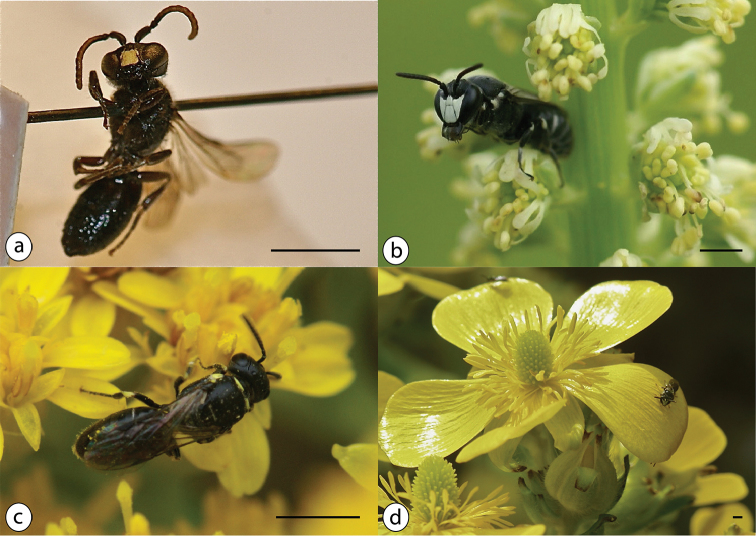
Azorean *Hylaeus* species: **a** *Hylaeus azorae* male, Holotype from Pico, Oberösterreichisches Landesmuseum Linz **b** *Hylaeus signatus* male in *Reseda luteola* (Resedaceae), regurgitating nectar, June 2012, Graciosa **c** *Hylaeus pictipes* female, dorsal view, on *Solidago azorica* (Asteraceae), July 2015, Corvo **d** *Hylaeus pictipes* male (second male in the background) on *Ranunculus cortusifolius* (Ranunculaceae), June 2012, Corvo; photos: Hanno Schaefer (**a, b, d**); Julie A. Weissmann (**c**); scale bars 2 mm.

Male 6–7 mm, female unknown; sternite 7 with winged appendages, sternite 8 deeply V-shaped (Fig. 6a).

###### Distinguishing features.

Male with +/- rectangular yellowish face pattern (Fig. 5a); female unknown.

###### General distribution.

Endemic to the Azores.

###### Distribution in the Azores.

Pico, Montanha do Pico.

###### First record.

1938 (described in Warncke 1992b, known only from the type).

###### Nesting.

Unknown, probably in hollow twigs.

###### Social behaviour.

Unknown, but probably solitary.

###### Foraging.

Unknown.

###### Phenology.

July.

###### Material.

Pico, 10.07.1938, 1 male, leg. Ragnar Storå, coll. Linz.

##### Hylaeus (Prosopis) pictipes

Taxon classificationAnimaliaHymenopteraColletidae

(Nylander)

###### Description.

Small black bee (wing length 3.5 mm, total length c. 5 mm) with elongate face, yellow face patterns, and black mandibles (Fig. 5b, d, 6c); groove (fovea) alongside inner eye margin curved towards lateral ocelli (cf. Falk and Lewington 2015, p. 81).

###### Distinguishing features.

Smallest *Hylaeus* in the Azores, male with yellow face pattern exceeding insertion point of antennae, female face pattern with two small trapezoidal yellow spots; legs of males mainly yellow.

###### General distribution.

Eurasia, from Portugal to the Caucasus.

###### Distribution in the Azores.

Corvo, Faial, Flores, Graciosa, Pico, São Miguel.

###### First record.

1938 (first mentioned in Warncke 1992b).

###### Nesting.

Builds nests in pithy stems or uses existing cavities (e.g., hollow twigs, holes of wood-boring beetles).

###### Social behaviour.

Solitary.

###### Foraging.

Polylectic, observed on Ranunculaceae (*Ranunculus cortusifolius*) (Fig. 6d) and Asteraceae (*Solidago azorica*, *Helminthotheca echioides*).

###### Phenology.

June-August.

###### Material.

Faial (Horta), 30.06.–05.07.1938, 1 female, leg. Richard K. H. Frey; Pico (Madalena), 6–9.07.1938, 1 male, leg. Ragnar Storå; Pico (Madalena), 6–9.07.1938, 1 female, leg. Richard K. H. Frey; S. Miguel (San Roque), 21.07.1938, 1 male, leg. Richard K. H. Frey, all det. and coll. Warncke (Linz). Corvo, 3 males; Graciosa (rim of Caldeira), 1 male; Pico (São Roque), 2 females, all leg. H. Schaefer, coll. TUM (B3-B7, B44).

The COI sequence of specimen H. Schaefer B6 (TUM), acc. no. KX824777, differs in 1–7 positions from the five *Hylaeus pictipes* sequences from Spain and Germany in GenBank (see Fig. 2).

##### Hylaeus signatus

Taxon classificationAnimaliaHymenopteraColletidae

(Panzer)

###### Description.

Medium-sized black bee (total length 7–9 mm in both sexes, wing length 4.5–5.5 mm in females and 4–6 mm in males) with roundish face and conspicuous yellowish-white face markings, without curved grooves (fovea) along eye margin; mandibles and legs black; white hair bands on lateral sides of tergite 1 (Fig. 6b).

###### Distinguishing features.

Relatively large; male with mask not exceeding insertion point of antennae (Fig. 5 c), female face pattern with the two spots larger than in *Hylaeus pictipes* and more triangular in outline (Fig. 5e); *Reseda* specialist.

###### General distribution.

Madeira (introduced); North Africa; Eurasia, from Portugal in the West to northern Scandinavia and Uzbekistan in the East.

###### Distribution in the Azores.

All islands except São Jorge.

###### First record.

2001 (Santa Maria; H. Schaefer, unpublished data).

###### Nesting.

In existing cavities (twigs, earth, abandoned nests of other hymenoptera, etc.), sometimes in dense aggregations.

###### Social behaviour.

Solitary bee.

###### Foraging.

Oligolectic, usually *Reseda* specialist, but in the Azores also observed on *Tamarix africana* (Tamaricaceae).

###### Phenology.

June-August.

###### Material.

Corvo (Vila do Corvo), 04.08.2014, 1 male; Graciosa, June 2012, 3 females, 7 males on *Tamarix*; Terceira (airport), 08.08.2014, 1 female, 1 male; Santa Maria, 1 female, all leg. H. Schaefer, coll. TUM (B8-B20; B45).

The COI sequence of specimen H. Schaefer B12 (TUM), acc. no. KX824778, is identical to a *Hylaeus signatus* sequence from Germany (KJ837965, see Fig. 2).

### Halictidae

#### *Halictus* Latreille

**Figure 7.**
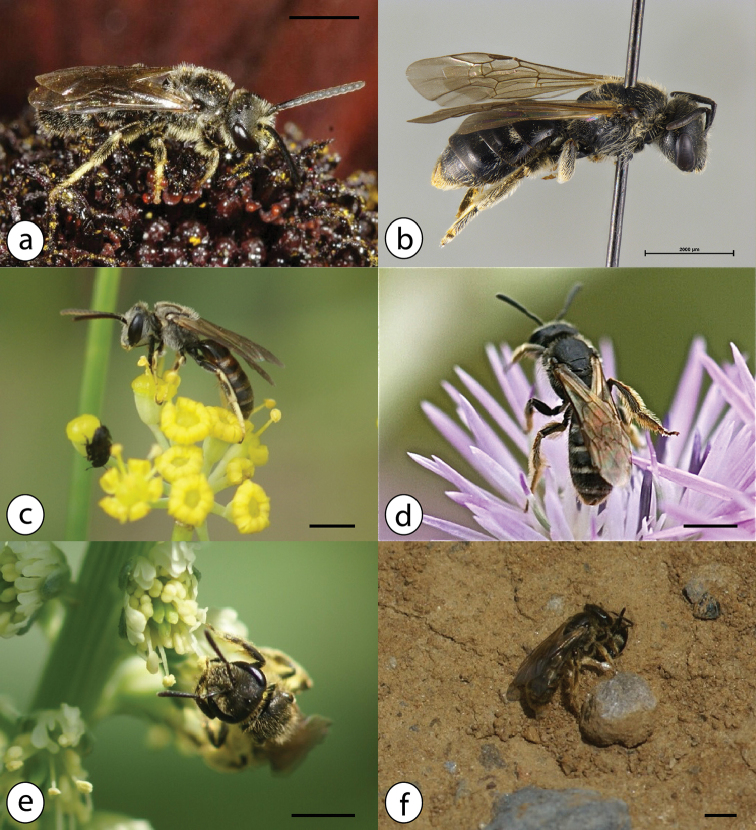
Azorean *Halictus* species: **a** *Halictus lativentris* male, UK **b** *Halictus lativentris* female from Faial, leg. P. Wirtz **c** *Halictus malachurus* male on *Foeniculum vulgare* (Apiaceae) with *Brassicogethes* beetle in the foreground, July 2016, Corvo **d** *Halictus malachurus* female on *Galactites tomentosus* (Asteraceae), June 2012, Graciosa; **e** *Halictus malachurus* female on *Reseda luteola* (Resedaceae), July 2016, Corvo **f** *Halictus malachurus* female approaching nest, while another female guards the entrance hole and touches the antennae of the arriving bee to control its scent, July 2016, Corvo; photos: Jeremy Early (**a**), Esther Ockermüller (Oberösterreichisches Landesmuseum Linz) (**b**), Julie A. Weissmann (**c, e, f**); Hanno Schaefer (**d**); scale bars 2 mm.

All Azorean *Halictus* species are small brownish bees, often occurring in large numbers and nesting in the ground. Males are elongate bees with long antennae (Fig. 7a). Females of the genus can be recognized by a median furrow on the otherwise hairy tergite 5 near the tip of the abdomen (Fig. 7d) and relatively short antennae. Females collect pollen with help of pollen brushes on their hind legs. Both solitary and eusocial species of this genus exist in the Azores. The Azorean species are commonly treated as members of the genus *Lasioglossum* but we follow Amiet and Krebs (2014) and include *Lasioglossum* in a broadly circumscribed *Halictus*. Six species in the Azores.

##### Halictus lativentris

Taxon classificationAnimaliaHymenopteraHalictidae

(Schenk)

Halictus lativentris (Schenk), (Lasioglossum lativentre)

###### Description.

Small, dark brown bee (total length 8–9 mm in both sexes, wing length 5,5–6 mm in females and 4,5–5,5 mm in males), with narrow bands of pale hair on the abdomen; stigma light brown (Fig. 7a–b).

###### Distinguishing features.

Unknown.

###### General distribution.

West- and Central Europe.

###### Distribution in the Azores.

Faial, São Miguel.

###### First record.

1984 (first mentioned by Stöckl 1988).

###### Nesting.

Probably in light soils.

###### Social behaviour.

Solitary.

###### Foraging.

No observation data from the Azores available, probably polylectic.

###### Phenology.

July-August.

###### Material.

São Miguel (Remédios), 10.08.1984, 1 male, leg. La Roche, det. Ebmer (Ebmer, pers. comm., 20.09.2016); Faial (Horta), 22.07.1992, 1 female, leg. P. Wirtz, det. Ebmer, coll. Warncke (Linz).

##### Halictus malachurus

Taxon classificationAnimaliaHymenopteraHalictidae

(Kirby)

Halictus malachurus (Kirby), (Lasioglossum malachurum)

###### Description.

Small dark brown bee (total length 7–10 mm in both sexes, wing length 5,5–6,5 mm in females and 4,5–6,5 mm in males); males and females with densely punctuated scutum and top of propodeum with angulate hind corner; males with black or extensively red abdomen, particularly long antennae and extensively yellow legs (Fig. 7c–f).

###### Distinguishing features.

The largest representative of the genus in the Azores; “shoulders” of the thorax strongly angled (Fig. 7e).

###### General distribution.

North Africa; Eurasia, from Portugal to Iran and North to Denmark.

###### Distribution in the Azores.

All islands except Terceira (where probably overlooked).

###### First record.

1930 (first mentioned by Benoist et al. 1936).

###### Nesting.

Ground nesting (mostly in compacted soil), often in large aggregations; sentinel bee closes access with her head (Fig. 7f).

###### Social behaviour.

Eusocial, sometimes polygynous, and sometimes (in warmer environments) with several broods of workers.

###### Foraging.

Polylectic, observed e.g., on Apiaceae (*Foeniculum vulgare*), Asteraceae (*Galactites tomentosus*, *Helminthotheca echioides*, *Solidago azorica*, *Tolpis azorica*) and Resedaceae (*Reseda luteola*).

###### Phenology.

July-September.

###### Material.

São Miguel (Furnas) and Faial (Horta), August-September 1930, leg. L. Chopard, det. Blüthgen (Benoist et al. 1936, not seen). São Miguel, September 1954, 5 males, leg. M. Diñiz. São Miguel (Lagoa das Furnas), 07.08.1984, 1 female; São Miguel (Ponta Delgada), 12.08.1984, 2 females; São Miguel (Ginetes), 11.08.1984, 2 females; São Miguel (São João), 09.08.1984, 2 females, 2 males; São Miguel (Feteiras), 11.08.1984, 4 males, all leg. La Roche, det. Ebmer (Ebmer, pers. comm.). Flores, 02.08.2014, 1 female; Graciosa, 3 females, 1 male, all leg. H. Schaefer, coll. TUM (specimens B21-B25).

The COI sequences of specimens H. Schaefer B21 and B23 (TUM), acc. no. KX824763-64, differ in 1–5 positions from the *Halictus malachurus* sequences from Spain and Germany in GenBank (see Fig. 2).

##### Halictus minutissimus

Taxon classificationAnimaliaHymenopteraHalictidae

(Kirby)

Halictus minutissimus (Kirby), (Lasioglossum minutissimum; Evylaeus minutissimus)

###### Description.

**Figure 8.**
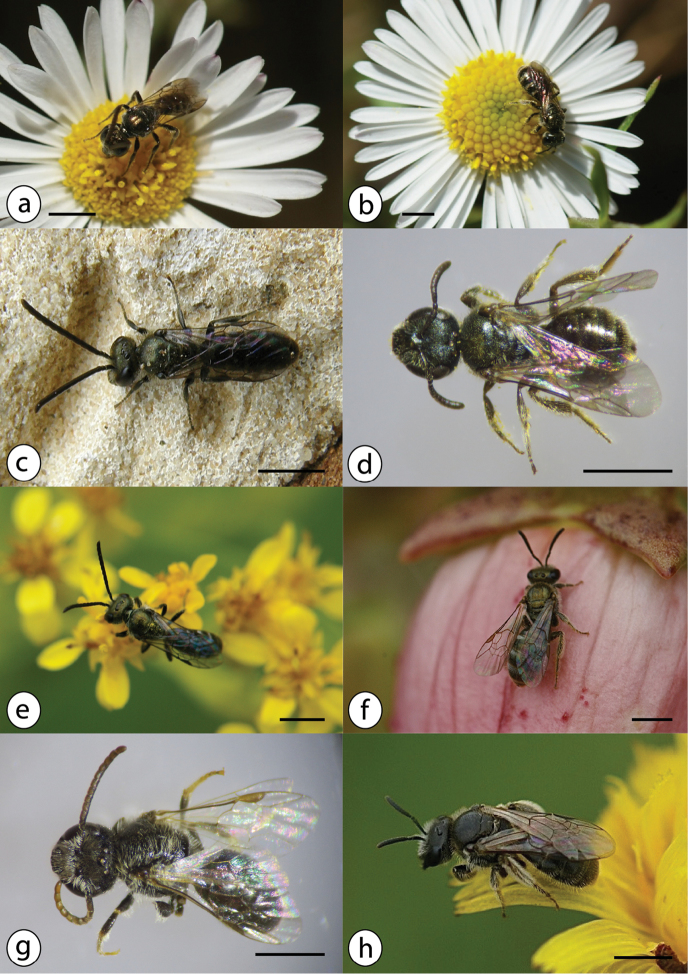
Azorean *Halictus* species (continued): **a** *Halictus minutissimus* male on *Erigeron karvinskianus* (Asteraceae), July 2016, Terceira (Monte Brasil) **b** *Halictus minutissimus* female on *Erigeron karvinskianus* (Asteraceae), July 2016, Terceira (Monte Brasil) **c** *Halictus morio* male, UK **d** *Halictus morio* female from Graciosa (B29, TUM) **e** *Halictus smeathmanellus* male on *Solidago azorica* (Asteraceae), August 2015, Flores **f** *Halictus smeathmanellus* female on *Azorina vidalii* (Campanulaceae), July 2015, Corvo **g** *Halictus villosulus* male from Corvo (B60, TUM) **h** *Halictus villosulus* female on *Leontodon taraxacoides* (Asteraceae), June 2012, Graciosa; photos: Julie A. Weissmann (**a, d, e, f, g**); Hanno Schaefer (**b, h**); Steven Falk (**c**); scale bars 2 mm.

Tiny blackish bee (total length c. 5 mm in both sexes, wing length 3,5–4 mm in females and 3–3,5 mm in males); males with entirely dark hind legs (Fig. 8a–b).

###### Distinguishing features.

Smallest of the *Halictus* species in the Azores, behaviour more hectic than other bees.

###### General distribution.

West- and Central Europe.

###### Distribution in the Azores.

Faial, São Miguel, Terceira.

###### First record.

1986 (first mentioned by Ebmer 1988).

###### Nesting.

Ground nests, sometimes in large aggregations.

###### Social behaviour.

Solitary.

###### Foraging.

Polylectic, observed on Asteraceae (*Erigeron karvinskianus*).

###### Phenology.

July.

###### Material.

Faial (Horta), July 1986, 1 female, leg. Aptroot, det. Ebmer, coll. Mus. Leiden (Ebmer 1988); São Miguel (Ponta Delgada), July 1992, leg. Wirtz, det. Warncke (Wirtz 1994, not seen).

##### Halictus morio

Taxon classificationAnimaliaHymenopteraHalictidae

(Fabricius)

Halictus morio (Fabricius), (Lasioglossum morio)

###### Description.

Small black bee (total length 7–8 mm, wing length 4 mm), in the sun often shining metallic green; with narrow bands of whitish hair on the abdomen (Fig. 8c–d).

###### Distinguishing features.

With *Halictus smeathmanellus*, one of the two small and greenish representatives of the genus in the Azores, but smaller in size than the former species; thorax and abdomen metallic green; males with entirely black tarsi.

###### General distribution.

North Africa; Eurasia, from Portugal to Kazakhstan and north to Finland.

###### Distribution in the Azores.

All islands except Faial (where probably overlooked).

###### First record.

1930 (first mentioned by Benoist et al. 1936).

###### Nesting.

On bare or sparsely vegetated surfaces on south-facing slopes or flat areas (here, the entrance often with small conical tumuli), often in large aggregations.

###### Social behaviour.

Primitively eusocial.

###### Foraging.

Polylectic, observed on Asteraceae (*Helminthotheca echioides*, *Sonchus asper*) and Oxalidaceae (*Oxalis corniculata*).

###### Phenology.

April-September.

###### Material.

São Miguel (Furnas), August-September 1930, leg. L. Chopard, det. Blüthgen (Benoist et al. 1936, not seen). São Miguel (2 km SE Feteiras), 11.08.1984, 3 females 1 male; São Miguel (Feteiras), 11.08.1984, 3 females, 2 males; São Miguel (Santana), 07.08.1984, 1 male; São Miguel (Ponta Delgada), 12.08.1984, 1 male, São Miguel (Vila Franca), 07.08.1984, 1 male, all leg. La Roche, det. Ebmer (Ebmer, pers. comm.). São Miguel (Lagoa das Furnas), 23.04.1984, 1 male, leg. Suko, det. Ebmer, coll. Mus. Bremen (Ebmer, pers. comm.). São Miguel (Ponta Delgada), July 1992, leg. Wirtz, det. Warncke (Wirtz 1994, not seen). Santa Maria (Vila do Porto), July 1972, 1 female, det. Ebmer, coll. Senckenberg Frankfurt (Ebmer, pers. comm.). Graciosa (Serra Branca), 4 females; Graciosa (Serra dos Fontes), 3 females; Terceira, 08.08.2014, 1 female, all leg. H. Schaefer, coll. TUM (specimens B26-B32, B50).

The COI sequences of specimens H. Schaefer B27 and B50 (TUM), acc. no. KX824760-61, differ in 1–2 positions from the closest *Halictus morio* sequences in GenBank, which are of German origin (see Fig. 2).

##### Halictus smeathmanellus

Taxon classificationAnimaliaHymenopteraHalictidae

(Kirby)

Halictus smeathmanellus (Kirby), (Lasioglossum smeathmanellum)

###### Description.

Small black bee (wing length 4.5 mm in females and 4–4.5 mm in males), in the sun shining metallic green, scutum sparsely punctuated (Fig. 8e–f).

###### Distinguishing features.

With *Halictus morio*, one of the two small and greenish representatives of the genus in the Azores, but larger in size than the former species.

###### General distribution.

North Africa (Morocco); in Europe from the Iberian Peninsula to Scotland and SW Germany.

###### Distribution in the Azores.

Corvo, Flores, Pico, São Miguel, Terceira.

###### First record.

First mentioned in Blüthgen (1944), no island specified.

###### Nesting.

Mostly in vertical structures (sparsely vegetated slopes, crevices in cliffs or walls), often in large aggregations.

###### Social behaviour.

Probably solitary (http://www.bwars.com/bee/halictidae/lasioglossum-smeathmanellum) but no observations from the Azores available.

###### Foraging.

Polylectic, observed on Asteraceae (*Solidago azorica*).

###### Phenology.

July-August.

###### Material.

Pico (Madalena), July 1986, 6 females, 1 male, det. Ebmer, coll. Museum Leiden (Ebmer, pers. comm.). Terceira (airport), 1 female, leg. H. Schaefer, B36 (TUM).

The COI sequence of specimen H. Schaefer B36 (TUM), acc. no. KX824762, is identical to a *Halictus smeathmanellus* sequence from England in GenBank (KT074061, see Fig. 2).

##### Halictus villosulus

Taxon classificationAnimaliaHymenopteraHalictidae

(Kirby)

Halictus villosulus (Kirby), (Lasioglossum villosulum)

###### Description.

Medium-sized bee (total length 6–9 mm in females and 6–8 mm in males, wing length 4.5–5.5 mm in females and 4–4.5 mm in males,); scutum with unusually large punctures, top of propodeum roundish; head and thorax hairy; abdomen shiny black (Fig. 8g–h).

###### Distinguishing features.

Medium-sized *Halictus* (similar in size to *Halictus smeathmanellus* but not metallic green); head densely white villous, thorax and abdomen blackish dark with scattered long white hairs.

###### General distribution.

Madeira, Canaries; North Africa; Eurasia, from Portugal to Nepal and Malaysia in the East and Finland in the North.

###### Distribution in the Azores.

All islands.

###### First record.

1930 (first mentioned by Benoist et al. 1936).

###### Nesting.

In various substrates on sparsely vegetated slopes, cliffs or flat areas (in the latter, the entrance often with small tumuli); often in large aggregations.

###### Social behaviour.

Solitary.

###### Foraging.

Polylectic, observed on Asteraceae (*Helminthotheca echioides*, *Hypochaeris radicata*, *Leontodon taraxacoides*, *Sonchus asper*) and Frankeniaceae (*Frankenia laevis*).

###### Phenology.

June-September; *Halictus villosulus* is known to be bivoltine (two generations per year) elsewhere but no detailed observational data is available from the Azores.

###### Material.

São Miguel (Furnas) and Terceira (Monte Brasil), August-September 1930, leg. L. Chopard, det. Blüthgen (Benoist et al. 1936, not seen). São Miguel (Ponta Delgada), July 1992, leg. Wirtz, det. Warncke (Wirtz 1994, not seen). São Miguel (Ponte), July 1986, 3 females; Faial (Horta), July 1986, 5 females, 5 males; Pico (Magdalena), July 1986, 1 male, all det. Ebmer, coll. Museum Leiden (Ebmer, pers. comm.). São Miguel (S. João), 09.08.1984, 1 female, 1 male; São Miguel (Ponta Delgada), 12.08.1984, 1 female; São Miguel (Vila Franca), 07.08.1984, 1 male; São Miguel (Remedios), 10.08.1984, 1 male, all leg. La Roche, det. Ebmer (Ebmer, pers. comm.). Santa Maria (Vila do Porto), July 1972, 2 females, det. Ebmer, coll. Senckenberg Frankfurt (Ebmer, pers. comm.). Corvo (lighthouse), 5 females, 1 male; Flores (Ponta da Faja), 05.08.2014, 3 females; Graciosa, June 2012, 6 females; Terceira (airport), 1 male; Terceira, 08.08.2014, 1 female, all leg. H. Schaefer, coll. TUM (specimens B33-B35, B37-B43, B51, B57-B62).

The COI sequence of specimens H. Schaefer B33, B38, B51, B60-B62 (TUM), acc. no. KX824765-70, are (near) identical to a *Halictus morio* sequence from France in GenBank (JF903563), but they differ in at least 15 positions from the remaining *Halictus morio* sequences in GenBank (see Fig. 2).

#### Megachilidae

The females of all representatives of this group collect pollen with a pollen brush beneath their abdomen.

#### *Anthidium* Fabricius

The genus is characterised by a specialised nesting behaviour: females build their nests from plant hairs or rarely resin and can be recognised when transporting such balls of plant fibres or resin in their mandibles. The males show territorial behaviour. Solitary. One species with limited distribution in the Azores, likely an introduction.

##### Anthidium manicatum

Taxon classificationAnimaliaHymenopteraMegachilidae

(Linnaeus)

###### Description.

**Figure 9.**
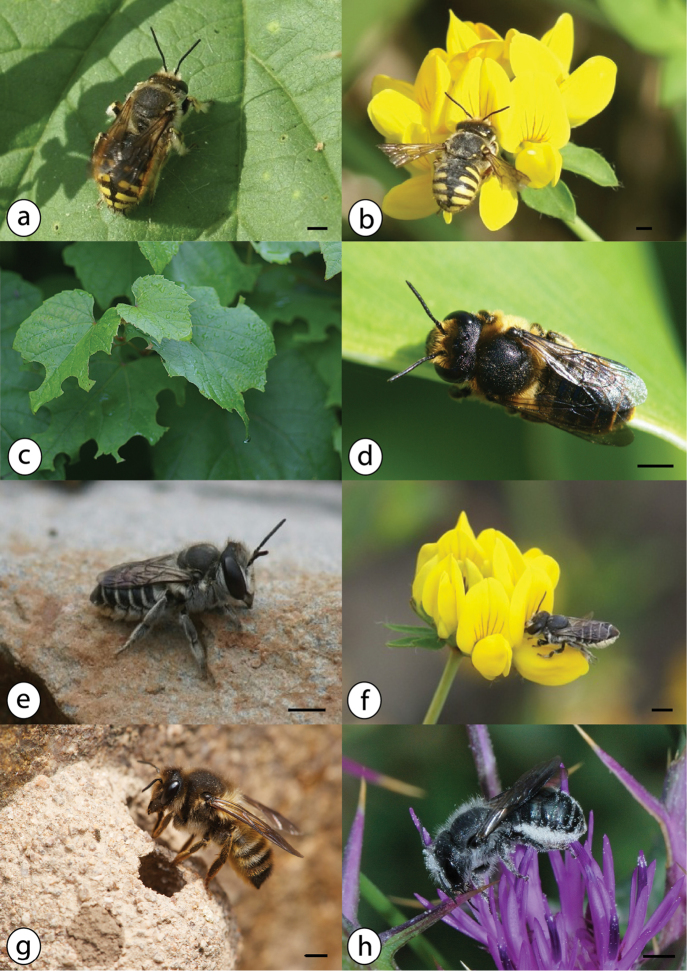
Azorean Megachilidae: **a** *Anthidium manicatum* male, note the spine-like extensions at the tip of the abdomen, July 2016, Faial **b** *Anthidium manicatum* female on *Lotus corniculatus* (Fabaceae), July 2016, Faial **c** grapevine, *Vitis vinifera* (Vitaceae), leaves with oval and circular cuts made by leafcutter bees, probably *Megachile centuncularis*, June 2013, Corvo **d** *Megachile centuncularis* female resting on leaf of Kahili ginger, *Hedychium gardnerianum* (Zingiberaceae), August 2015, Flores **e** *Megachile concinna* female, July 2016, Flores **f** *Megachile concinna* female on *Lotus corniculatus* (Fabaceae), July 2016, Corvo **g** *Megachile pyrenaica*/*rufescens*, female on its clay nest, 26.04.2010, France (Le Muy) **h** *Osmia niveata*, female on *Centaurea* spec., Israel; photos: Julie A. Weissmann (**a, b, d, e, f**), Hanno Schaefer (**c**), Nico J. Vereecken (**g**), Gideon Pisanty on wikipedia (creative commons license) (**h**); scale bars 2 mm.

Total length 11–12 mm in females and 14–18 mm in males, wing length 8–10 mm in females and 9,5–12 mm in males; wings dark, face and legs yellow, body black with yellow spots and bands, tergite 7 of males with five black spine-like extensions (Fig. 9a–b).

###### Distinguishing features.

Both sexes with conspicuous yellow-black abdominal patterns; can be confused only with similar looking syrphid flies or species of wasps, especially the introduced *Vespula germanica*, but differs from all these taxa in flight behaviour and abdominal pollen collection (in females).

###### General distribution.

Eurasia, North Africa; introduced in the Canaries, North and South America, New Zealand.

###### Distribution in the Azores.

Faial, São Miguel.

###### First record.

1857 (Drouët 1861).

###### Nesting.

In existing holes; cell walls and closing plugs are built out of plant fibres.

###### Social behaviour.

Solitary.

###### Foraging.

Polylectic; preference for Lamiaceae, where the females also collect plant hairs for their nest, observed on *Lotus corniculatus* (Fabaceae).

###### Phenology.

July-September.

###### Material.

Faial (Horta), September 1952, 1 female (Carthy 1955, not seen). São Miguel (Ponta Delgada), July 1992, leg. Wirtz, det. Warncke (Wirtz 1994, not seen).

#### *Megachile* Latreille

The genus is known for two different types of specialised nesting behaviour (Trunz et al. 2016). In subgenus *Megachile*, the female bees build their nests in holes of wood or stone and use pieces of cut-out sections of leaves or petals (“leaf-cutter bees”). Their cuttings are quite conspicuous (Fig. 9c) and can help to locate the bees in the field. In subgenus *Chalicodoma*, females construct nests of mud and sand instead of leaf pieces (Fig. 9g). Females of the genus are easy to recognize by their strong mandibles and by their tendency to bend the abdomen upwards when visiting flowers. Solitary. Three species in the Azores.

##### Megachile centuncularis

Taxon classificationAnimaliaHymenopteraMegachilidae

(Linnaeus)

###### Description.

Medium-sized (total length 11–12 mm in females and 9–11 mm in males, wing length 7–8,5 mm in females and 7–8 mm in males) dark brown bees with yellowish hair and reddish abdominal pollen collecting brushes (Fig. 9d).

###### Distinguishing features.

Females with conspicuous ventral pollen brushes and strong mandibels.

###### General distribution.

West- and Central Europe.

###### Distribution in the Azores.

Corvo, Faial, Flores, São Jorge, São Miguel, Terceira.

###### First record.

1865 (Godman 1870).

###### Nesting.

In existing holes (e.g. in dead wood, walls, soil or twigs); cell walls and closing plugs are built of leaf sections.

###### Social behaviour.

Solitary.

###### Foraging.

Polylectic, Asteraceae, Fabaceae.

###### Phenology.

July-September.

###### Material.

São Miguel (Furnas, Ponta Delgada), August-September 1930, leg. L. Chopard, det. Benoist (Benoist et al. 1936, not seen). Faial (Horta), August-September 1952, 2 females (Carthy 1955, not seen). Terceira, 2 females, leg. A. Picanço MF22.1, MF22.2 (EDTP).

The COI sequences of specimens MF22.1, MF22.2 (EDTP), acc. no. KX824779-80, are identical to a *Megachile centuncularis* sequence from Germany in GenBank (KJ838449), and very similar to others from Canada, Croatia, and Germany (see Fig. 2).

##### Megachile concinna

Taxon classificationAnimaliaHymenopteraMegachilidae

Smith

Megachile concinna Smith; syn.: Megachile atratula Rebmann (see Gonzalez et al. 2010)

###### Description.

Smallest of the leafcutter bees in the Azores (total length 9 mm in females and 7 mm in males). Strongly banded tergites, silvery-brown appearance. Females have a silvery pollen brush underneath the abdomen (Fig. 9e).

###### Distinguishing features.

Smallest of the leafcutter bees on the Azores, silvery-brown appearance; fast flyer.

###### General distribution.

Mediterranean region.

###### Distribution in the Azores.

Corvo, Flores, Faial.

###### First record.

Ornosa et al. (2007), as *Megachile atratula*.

###### Nesting.

Probably in sandy ground near the coast but no nest observations available from the Azores.

###### Social behaviour.

Solitary.

###### Foraging.

Polylectic, with strong preference for Fabaceae, especially *Lotus* species (Fig. 9f).

###### Phenology.

Observed in July-August.

###### Material.

None seen; listed in the AMNH database for the Azores - http://www.discoverlife.org/mp/20l?id=AMNH_BEES67697

###### Note.

The *Megachile concinna* complex is currently focus of phylogenetic analyses by C. Praz but no Azorean material was included in the study (C. Praz, pers. comm., Sept. 2016).

##### Megachile pyrenaica

Taxon classificationAnimaliaHymenopteraMegachilidae

Lepeletier

Megachile pyrenaica Lepeletier, (Chalicodoma pyrenaicum)

###### Description.

Medium-sized (total length 13–16 mm), dark, very hairy bees (Fig. 9g).

###### Distinguishing features.

Only bee species in the Azores that builds large clay nests.

###### General distribution.

Mediterranean region.

###### Distribution in the Azores.

Santa Maria.

###### First record.

2012 (see: http://www.gba.uac.pt/media/press&events/ver.php?id=126)

###### Nesting.

Unknown.

###### Social behaviour.

Solitary.

###### Foraging.

Probably oligolectic on Fabaceae (Scheuchl and Willner 2016) but no observational data available from the Azores.

###### Phenology.

June-July.

###### Material.

Santa Maria (near airport), 2 specimens, 2012, det. Kratochwil, coll. Kratochwil (pers. comm., 30.09.2016).

###### Note.

Easily confused with the morphologically very similar *Colletes rufescens*, from which it differs mainly by its orange tarsi (M. Aubert, pers. comm., Sept. 2016).

#### *Osmia* Panzer

Build their nest in existing cavities in wood or stone. Solitary. One species in the Azores but no recent confirmation.

##### Osmia niveata

Taxon classificationAnimaliaHymenopteraMegachilidae

(Fabricius)

Osmia niveata (Fabricius), syn. Osmia fulviventris (Panzer)

###### Description.

Small dark bee (total length 8–10 mm; wing length 7–8 mm in females and 6–7 mm in males) with conspicuous orange red pollen collecting brushes on the underside of the abdomen (Fig. 9h).

###### General distribution.

Madeira, West Palaearctic.

###### Distribution in the Azores.

Probably São Miguel and Terceira (see below).

###### First record.

1865 (Godman 1870).

###### Nesting.

Probably in existing cavities in wood or stone but no observations from the Azores available.

###### Social behaviour.

Solitary.

###### Foraging.

Oligolectic on Asteraceae, with preference for thistles and relatives (Scheuchl and Willner 2016) but no foraging observations from the Azores available.

###### Phenology.

Unknown.

###### Material.

No specimens seen/known to us.

###### Note.

Records of *Osmia emarginaria* St. Farg from São Miguel and Terceira (Godman 1870) most likely refer to this species.

### Domesticated species

#### *Apis* Linnaeus

##### Apis mellifera

Taxon classificationAnimaliaHymenopteraApidae

Linnaeus

###### Description and distinguishing features.

**Figure 10.**
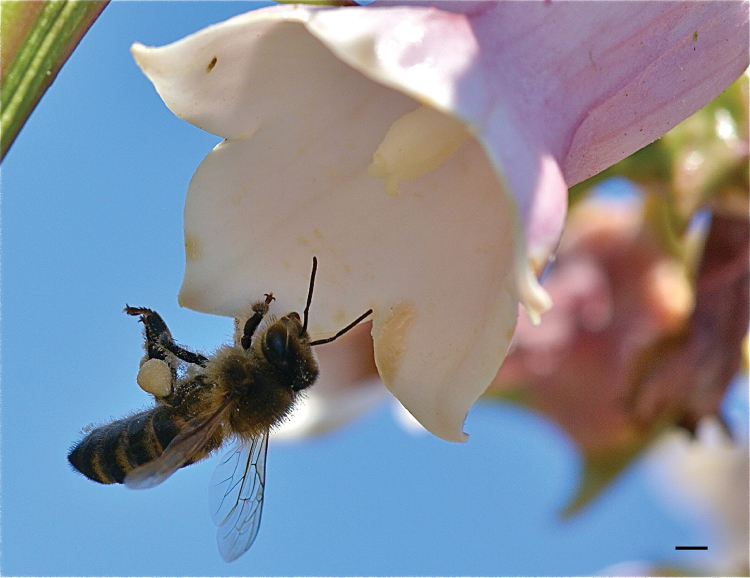
*Apis mellifera* worker on *Azorina vidalii* (Campanulaceae), August 2015, Flores; photo: Julie A. Weissmann; scale bar 2 mm.

Honeybees are larger than most wild bees in the islands (total length 11–13 mm in workers, 13–16 mm in males; wing length 9–10 mm in workers and 12–13,5 mm in males) but can be confused with some syrphid flies (e.g., *Eristalis tenax*). They differ from superficially similar *Megachile* by their pollen-collecting mode: scopae on the legs (Fig. 10), not abdominal brushes.

###### General distribution.

Probably native to the Mediterranean region but kept by beekeepers in temperate regions worldwide for honey production.

###### Distribution in the Azores.

Apiculture is declining in the Azores (maybe as a result of accidental introduction of *Varroa destructor* mites) but honeybee colonies are still kept on all islands; in 2010, the number of bee colonies in the Azores was c. 3850, belonging to 255 beekeepers (source: Paulo Miranda at http://montedomel.blogspot.de).

###### First record.

16^th^ century (Frutuoso 2005); according to Marques (cited in Crane 1999: 219) “[b]eekeeping was started in 1554 (…), the bees probably being taken there from Portugal.”

###### Nesting.

Today, honeybees are not known to occur as escaped/feral bees in the archipelago but Drouët (1861) reported wild honeybees from São Miguel.

###### Social behaviour.

Highly eusocial.

###### Foraging.

Polylectic.

###### Phenology.

All year.

###### Material.

São Miguel (Furnas) and Terceira (Monte Brasil), August-September 1930, leg. L. Chopard, det. Benoist (Benoist et al. 1936, not seen).

###### Note.

De la Rúa et al. (2006) analysed genetic diversity of Azorean honeybees and found a comparably low number of mitochondrial haplotypes, some of them shared with Madeiran honeybees, other probably recent introductions from the mainland.

## Discussion

We add nine species to the latest list of Franquinho de Aguiar et al. (2010), which doubles the number of bee species in the archipelago. This massive increase is most likely not the result of numerous introduction or colonization events in the past few years but rather reflects the lack of research on Hymenoptera in the Azores (see also Borges et al. 2010). However, the origin of most species remains rather doubtful and some, e.g. *Bombus pratorum* and *Megachile pyrenaica*, are probably very recent introductions, which will perhaps not establish a permanent population and disappear after a few years.

The total number of now 18 wild bee species plus *Apis mellifera* is similar to Madeira, where Kratochwil et al. (2008) reported 15 species plus honeybee and Fellendorf et al. (1999) reported 18 species plus honeybee. In the Cape Verdes, the currently known species number is also similar (21 according to Báez et al. 2005, Straka and Engel 2012, Schaefer, unpubl. data). However, it is likely that more fieldwork in the Cape Verdes will lead to an increase of species numbers, especially in the halictid bees, where so far very few species are listed. In the well surveyed Canary Islands, bee diversity is much higher than elsewhere in Macaronesia, with 124 species, many of them with several subspecies (Báez et al. 2004). A comparison of species number per genus in the four archipelagos (Table 3) shows that the Canaries have a much higher number of genera, probably a result of the close proximity to the North African/Mediterranean bee diversity centre. Furthermore, several of them (e.g., *Andrena*, *Halictus*, *Osmia*) seem to have radiated in the Canaries, whereas no species rich bee radiations have been discovered so far in the remaining archipelagos.

**Table 3.** Comparison of species number per genus in the four Macaronesian archipelagos; for Madeira based on Fellendorf et al. (1999), for Canaries on Báez et al. (2004) and for Cape Verdes based on Báez et al. (2005), Straka and Engel (2012), and Schaefer, unpubl. data.

Genus	Azores	Madeira	Canaries	Cape Verdes
*Amegilla*	--	1	3	4
*Ammobates*	--	--	2	--
*Andrena*	--	3	21	--
*Anthidium*	1	--	2	--
*Anthophora*	--	--	4	--
*Apis*	[1]	[1]	[1]	[1]
*Bombus*	3	2	1	--
*Ceratina*	--	--	1	--
*Ceylalictus*	--	--	--	3
*Chiasmognathus*	--	--	--	1
*Colletes*	1	--	2	--
*Dasypoda*	--	--	1	--
*Dioxys*	--	--	2	--
*Dufourea*	--	--	2	--
*Epeolus*	--	--	1	--
*Eucera*	--	--	2	--
*Halictus* (incl. *Lasioglossum*)	6	3	16	2
*Heliophila*	--	--	3	--
*Hoplitis*	--	2	--	--
*Hylaeus*	3	2	5	--
*Megachile*	3	1	8	2
*Melecta*	--	--	5	--
*Melitta*	--	--	2	--
*Nomada*	--	--	5	--
*Nomioides*	--	--	4	2
*Osmia*	1	3	14	--
*Panurgus*	--	--	2	--
*Parammobatodes*	--	--	1	--
*Sphecodes*	--	--	6	1
*Stelis*	--	1	2	--
*Tetralonia*	--	--	4	--
*Thyreus*	--	--	3	4
*Xylocopa*	--	1	--	1
**Total species number** (excl. *Apis mellifera*)	**18**	**19**	**124**	**20**

When comparing bee species numbers per island in the Azores, it is important to keep in mind that our fieldwork on Pico, São Jorge, and São Miguel was less extensive than in the rest of the archipelago, so species numbers on these four islands are likely an underestimate of the true diversity. So far, it seems that bee diversity is fairly similar on all islands (seven to 14 species) and does not depend on classical biogeographical and ecological variables (island size, amount of natural vegetation, flowering plant diversity or altitude). More fieldwork is needed to confirm distribution per island and also assess altitudinal ranges and habitat preferences of the different bee species in the islands. There is recent evidence from Terceira that many bee species are foraging in disturbed habitats (Picanço et al., unpubl. data) but it is unclear if they can survive without natural vegetation. Further research on the impact of land use changes and the effects of invasive plant species on the long term survival of each of the Azorean bee species seems particularly important.

Five of the wild species (and the domesticated honeybee) are social, *Bombus pratorum*, *Bombus ruderatus*, *Bombus terrestris*, *Halictus malachurus*, and *Halictus morio*. The remaining species are solitary but three of them, *Halictus minutissimus*, *Halictus smeathmanellus*, and *Halictus villosulus* nest in aggregations. As pollen sources, most of the Azorean bees seem to visit a broad range of flowering plants. The only exception is *Hylaeus signatus*, which is mainly found on *Reseda* but not strictly specialized in the Azores, because it also visits *Tamarix africana*. None of the species in our list has a parasitic life form, which is remarkable given the high number of parasitic “cuckoo bees” on the mainland. The cuckoo bee *Sphecodes pseudofasciatus* Blüthgen (historically confused with *Sphecodes croaticus* Meyer) was listed for the Azores in the AMNH Bees database (http://www.discoverlife.org/mp/20l?id=AMNH_BEES44929), but this entry seems to be an error (J. Ascher, pers. comm., Sept. 2016). However, the occurrence of *Sphecodes* in the Azores would not be too surprising since several potential host species (halictids) exist and the genus is known from Cape Verde (Pauly et al. 2002, Straka and Engel 2012 [*Sphecodes capverdensis* = *Sphecodes pinguiculus*]) and the Canary Islands (Bogusch and Straka 2012, Warncke 1992a [*Sphecodes atlanticus*, *Sphecodes hirtellus*, *Sphecodes marginatus*, *Sphecodes punctipes*, *Sphecodes ruficrus*]) but apparently not from Madeira.

The Apidae of the Azores are another case where it seems important to caution against the uncritical use of checklist data. The number of bees in the Azores has been underestimated a lot so far. Moreover, while the latest checklist (Franquinho de Aguiar et al. 2010) classifies all bee species except the honeybee as “native”, none of them were seen as “natives” in the previous version by Borges et al. (2005). Currently, it seems best to classify only one species as endemic, the poorly known *Hylaeus azorae*, collected only once on Pico island but clearly morphologically distinct from any other Macaronesian *Hylaeus*. The related *Hylaeus pictipes* is found mainly in natural habitats on native plants and shows some genetic differentiation compared to mainland material. Therefore, we suggest to classify it as “possibly native” species, even if this is still a bit speculative. Based on the same reasoning, we also suggest to classify *Halictus villosulus* as “possibly native”, because it is mainly found in the higher altitudes of the islands in more natural vegetation than the other halictids and is also known from Madeira and the Canaries. For several of the remaining species, it seems clear that they are recent introductions because they are restricted to disturbed habitats at low altitudes, often on just a single island (e.g.: *Anthidium manicatum*, *Megachile pyrenaica*, *Hylaeus signatus*). Most of the halictid bees, of which endemic and native species are known in Madeira and the Canaries, are currently best classified as “probably introduced” until comprehensive phylogeographic analyses allow to make a more informed decision. The genetic data available so far, shows hardly any differentiation of the island populations from the mainland, which seems to reject the hypothesis of long isolated evolution following colonization prior to the arrival of human settlers in the 15^th^ century. The available material from the Azores also does not show any significant morphological differentiation from mainland populations (A. Ebmer, pers. comm.). Notably, we also do not find any evidence for an undescribed endemic *Halictus* species, which had been reported as “endemic super generalist” pollinator from Flores (Olesen et al. 2002). The widespread and probably introduced *Halictus malachurus* is today the most common bee in the study site of Olesen et al. (2002) on Flores and material collected by Olesen has been identified as *Halictus malachurus* (A. Ebmer, pers. comm.). This would suggest that contrary to the interpretation of Olesen et al. (2002), instead of endemic super generalists, an introduced pollinator complex, consisting mainly of *Halictus malachurus* and *Halictus morio* plus the less abundant *Halictus smeathmanellus* and *Halictus lativentris* might be most important for pollination networks in the Azores today. However, detailed observations, ideally on several islands and in different plant communities are needed before any definitive conclusion should be drawn.

From an evolutionary biology perspective, it is interesting that the percentage of endemic bees in the Azores (one endemic species, 5%) seems to be much lower than in Madeira (six endemic species, 33% (Fellendorf et al. 1999)) and the Canaries (46 endemic species, 37% (Báez et al. 2004)) or Cape Verdes (eleven endemic species, 55% (Báez et al. 2005, Straka and Engel 2012)). Whether this pattern is real and a result of e.g., the comparably young geological age or low habitat heterogeneity of the Azores, or simply reflects differences in the research intensity in the different archipelagos remains to be seen.

## Conclusions

With 18 wild species plus the domesticated honeybee, the bee diversity in the Azores seems similar to that of Madeira and Cape Verde but much lower than in the Canaries. The small number of endemic/native species suggests that the endemic flora of the Azores might have evolved mainly without presence of pollinating bees and adapted to other pollinator groups but more research is needed to confirm this hypothesis. More generally, we show that detailed taxonomic work and comprehensive fieldwork is needed before any checklist data can be used for macroecological studies (biodiversity comparisons) and ecological studies, e.g. pollination network analyses.

## Supplementary Material

XML Treatment for Bombus pratorum

XML Treatment for Bombus ruderatus

XML Treatment for Bombus terrestris

XML Treatment for Colletes eous

XML Treatment for Hylaeus (Prosopis) azorae

XML Treatment for Hylaeus (Prosopis) pictipes

XML Treatment for Hylaeus signatus

XML Treatment for Halictus lativentris

XML Treatment for Halictus malachurus

XML Treatment for Halictus minutissimus

XML Treatment for Halictus morio

XML Treatment for Halictus smeathmanellus

XML Treatment for Halictus villosulus

XML Treatment for Anthidium manicatum

XML Treatment for Megachile centuncularis

XML Treatment for Megachile concinna

XML Treatment for Megachile pyrenaica

XML Treatment for Osmia niveata

XML Treatment for Apis mellifera
